# *In vivo* monitoring of glial scar proliferation on chronically implanted neural electrodes by fiber optical coherence tomography

**DOI:** 10.3389/fneng.2014.00034

**Published:** 2014-08-21

**Authors:** Yijing Xie, Nadja Martini, Christina Hassler, Robert D. Kirch, Thomas Stieglitz, Andreas Seifert, Ulrich G. Hofmann

**Affiliations:** ^1^Neuroelectronic Systems, Department of General Neurosurgery, University Medical Center FreiburgFreiburg, Germany; ^2^Laboratory for Biomedical Microtechnology, Department of Microsystems Engineering(IMTEK), University of FreiburgFreiburg, Germany; ^3^Gisela and Erwin Sick Chair of Micro-optics, Department of Microsystems Engineering(IMTEK), University of FreiburgFreiburg, Germany

**Keywords:** chronic implants, flexible microelectrodes, foreign body reaction, optical coherence tomography, fiber catheter

## Abstract

In neural prosthetics and stereotactic neurosurgery, intracortical electrodes are often utilized for delivering therapeutic electrical pulses, and recording neural electrophysiological signals. Unfortunately, neuroinflammation impairs the neuron-electrode-interface by developing a compact glial encapsulation around the implants in long term. At present, analyzing this immune reaction is only feasible with post-mortem histology; currently no means for specific *in vivo* monitoring exist and most applicable imaging modalities can not provide information in deep brain regions. Optical coherence tomography (OCT) is a well established imaging modality for *in vivo* studies, providing cellular resolution and up to 1.2 mm imaging depth in brain tissue. A fiber based spectral domain OCT was shown to be capable of minimally invasive brain imaging. In the present study, we propose to use a fiber based spectral domain OCT to monitor the progression of the tissue's immune response through scar encapsulation progress in a rat animal model. A fine fiber catheter was implanted in rat brain together with a flexible polyimide microelectrode in sight both of which acts as a foreign body and induces the brain tissue immune reaction. OCT signals were collected from animals up to 12 weeks after implantation and thus gliotic scarring *in vivo* monitored for that time. Preliminary data showed a significant enhancement of the OCT backscattering signal during the first 3 weeks after implantation, and increased attenuation factor of the sampled tissue due to the glial scar formation.

## 1. Introduction

Intracortical electrodes are often employed in neuroprosthetic applications or in clinical stereotactic surgery, to deliver therapeutic electrical pulses and collect neuronal electrophysiological signals (Tronnier and Fogel, 2000; Stieglitz et al., 2009; Raspopovic et al., 2014). A reliable and dependable electrode-tissue-interface is crucial in this concept since a neuroprosthetic device is supposed to serve functionally over long period in subject's brain. Unfortunately, the lifetime of a such device is always compromised due to electrode corrosion or so called glial scarring around the indwelling electrode (Edell et al., 1992; Schmidt et al., 1993; Turner et al., 1999; Leach et al., 2010).

When an electrode is implanted into the brain, during the acute phase of the immune reaction, microphages settle in on the area and resident microglia cells of the immune system become activated and start proliferating. Then astrocytes are activated to scavenge the leftover large intruders. Unfortunately, any type of the artificial implant is resistant to this bio degradation, which induces a so called frustrated phagocytosis and leads in consequence to a dense glial scar attempting to isolate the implant from the delicate brain tissue (Turner et al., 1999; Biran et al., 2005; Polikov et al., 2005; Winslow et al., 2010; Potter et al., 2012). This isolation performed by glial sheathing not only changes the electrical coupling to the surrounding parenchyma, but may even cause neuronal loss by neurotoxic factors released from glial cells (Block et al., 2007). This leads to a deterioration and loss of electrophysiological signal over time. Electrical characterization of electrode-tissue state over time is attempted by temporally resolved impedance spectroscopy or signal-to-noise ratio analysis (Ludwig et al., 2006; Mcconnell et al., 2009). Immunohistochemical analysis is performed to characterize astrocytes and microglial cells progression over time. However, this requires tissue to be removed for postmortem examination disqualifying for long term *in vivo* process monitoring of the same animal (Szarowski et al., 2003). Lately, Kozai et al. (2012) reported using two-photon microscopy to reveal immediate microglial reaction to electrode insertion *in vivo*. However, this technique is limited to imaging only a shallow part (~200 μm) of the brain and only in short term (up to 7 h post implantation).

Alternatively, clinically approved Optical Coherence Tomography, displaying the intrinsic optical scattering properties of the tissue, might be used instead (Huang et al., 1991; Hee et al., 1996; Bonin et al., 2010). OCT features a spatial resolution in cellular range of 5–10 μm, up to 1.2 mm imaging depth in brain tissue, and up to 40,000 axial scans (A-scan) per second (Drexler et al., 2001; Rodriguez-Padilla et al., 2007). When integrated with ultra-small single mode fiber catheter, it is competent to visualize deep brain structures with minimum trauma (Tearney et al., 1997; Böhringer et al., 2006; Xie et al., 2013).

In the present study, we propose to use a fiber based spectral domain OCT to monitor the progression of the tissue's immune response through scar encapsulation progress in a rat animal model. We developed an integrated OCT detection probe consisting of an implantable ferrule based fiber catheter and a fiber patch cable. The fiber catheter was implanted in rat brain together with a flexible polyimide microelectrode array in sight, according to the procedure described in Richter et al. (2013), both intended to induce the brain tissue immune reaction. The OCT backscattering intensity of the brain tissue around the fiber increased during the first 3 weeks. The tissue optical attenuation coefficient altered after implantation and reached a maximum at the week 6 implying that a compact dense tissue sheath has developed around the fiber tip. Further more, the 10 μm thick flexible probe which located up to 2.7 mm away from the fiber catheter tip is still visible in the OCT image and A-scan plot.

## 2. Materials and methods

### 2.1. Optical coherence tomography imaging system

The imaging modality we used to monitor and assess the foreign body reaction is fiber based spectral domain optical coherence tomography (SDOCT) (Fercher et al., 1995). In this imaging modality, the incident light is transmitted into tissue through an implantable fiber catheter and the backscattered light from the illuminated tissue is also collected by the fiber catheter. The incident light and the backscattered light produce an interference pattern encoding the intrinsic optical properties of the sampled tissue. The interference pattern is detected at a spectrometer to construct A-scan (depth-scan) signals providing the backscattered light intensity as a function of depth.

**Figure 1:**
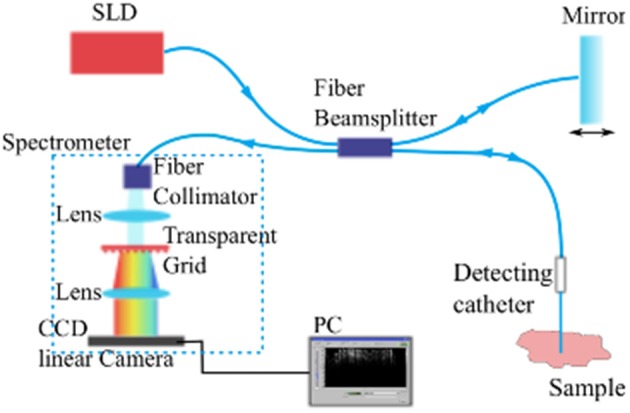
**A schematic diagram of the Spectral Domain Optical Coherence Tomography imaging system**.

The imaging system employed composes a commercially available fiber-based OCT module (“Callisto,” Thorlabs GmbH, Lübeck, Germany) utilizing a superluminescent diode (SLD) with center wavelength at 840 nm as the light source and a single mode fiber based sampling probe providing the feasibility of minimum-invasive imaging (see Figure 1). When equipped with a single mode fiber based detecting catheter, the whole system features an axial resolution of 14 μm and transversal resolution of 20 μm in air. The system's look-ahead capability is 3.5 mm from the tip of the fiber catheter to the tissue, enabling us to evaluate the development of the tissue reaction to the indwelling fiber catheter and to the flexible microelectrode underneath. It has been demonstrated that the fiber based OCT system is competent for imaging brain anatomical structures *in vivo* (Xie et al., 2013).

### 2.2. Optical fiber detection catheter

**Figure 2:**
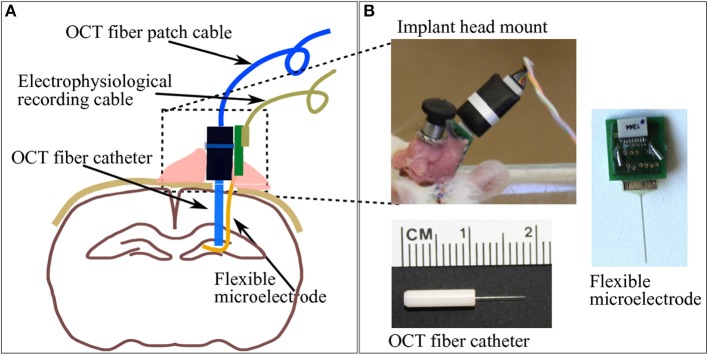
**(A)** A brief sketch of the implantations including a pair of indwelling probe: the OCT fiber catheter and the flexible polyimide microelectrode. **(B)** The picture on top-left shows the cemented fiber optics connector and PCB for electrophysiology recording taken on the second week after implantation surgery. Pictures on bottom-left and right demonstrate the geometry of the OCT fiber catheter and the flexible microelectrode connected to a PCB, respectively.

The aim of this study is to use a chronically implanted OCT fiber cannula to monitor and to assess brain tissue's foreign body reaction to it and to the also chronically implanted flexible microelectrode. The fiber cannula chosen consists of a ceramic FC ferrule (CF126-10, Thorlabs) with 2.5 mm outer diameter and 10.5 mm total length, and a 8 mm long single mode fiber (ø125 μm, SMF800-5.6-125, Thorlabs) in order to reach deep brain structures (see Figure 2B bottom for geometry).

### 2.3. Polyimide based flexible microelectrode

The flexible polyimide microelectrode, which also provokes immune response and which was implanted into the rat brain, is designed and fabricated in-house. The probe shank is 15 mm long, 10 μm thick and 365 μm wide, with a smooth surface. The substrate material of the photolithographically structured probe is a medical grade polyimide type PI2611 (U-Varnish S, UBE, Newyork), with a bulk YoungÕs modulus of around 10 GPa over 20 months in 37°C PBS (Rubehn and Stieglitz, 2010) (Figure 2B left). It is exemplifying a “device under test” based on findings (Sohal et al., 2014) that indwelling flexible substrates provoke a less pronounced gliosis compared with rigid micro electrodes presumably due to compliance-matching with brain tissue.

### 2.4. Animal surgery and device implantation

All animal experiments conducted in this study were performed with approval from the locally responsible Animal Welfare Committe with the Regierungspräsidium Freiburg in accordance with the guidelines of the European Union Directive 2010/63/UE. The animal model chosen consists of adult female Sprague Dawley rats (Charles River, Germany), weighing 280–320 g. The rats were initially anesthetized by administering intraperitoneally a mixture of 100 mg/kg ketamine and 5 mg/kg xylazine. Rats were placed on a the heating pad of a circulating water bath set to 40°C to maintain a body temperature of 35°C. The rat was then fixed into the stereotactic frame with a pair of ear pins and a bite bar. A 15 mm long scalp incision was cut along the midline, the skin was pulled aside to expose the skull surface structure. Bregma was identified and served as coordinate 0.0 mm. A burr hole (0.9 mm in diameter) in the skull at 4.0 mm posterior 2.0 mm lateral from Bregma was drilled. Prior to probe insertion, a small incision was cut on the dura and pia mater underneath the burr hole with a 27 G needle to avoid brain dimpling upon probe penetration.

**Figure 3:**
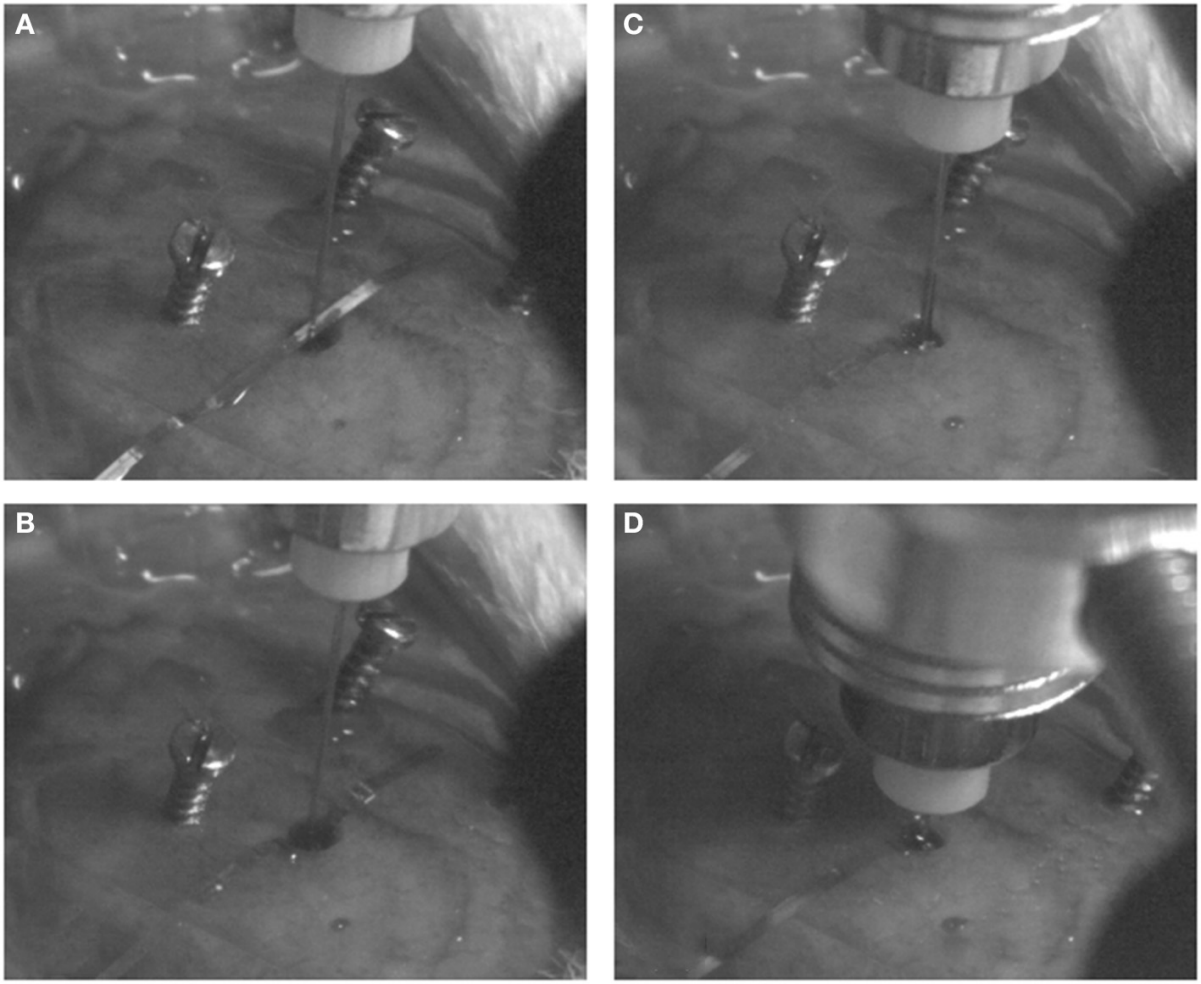
**Probe implantation procedures**. The flexible microelectrode and fiber catheter are precisely aligned above the burr hole **(A)**. The fiber catheter is slowly and cautiously driven down to push the shank of the flexible mciroelectrode into the brain **(B,C)** until the desired depth is reached **(D)**.

The flexible microelectrode was inserted in the brain tissue with the OCT fiber catheter, following the protocol prosposed by Richter et al. (2013). The flexible microelectrode was placed on the moist skull surface, with the shank precisely adjusted to overlay the burr hole at the position of 4 mm from the shank distal end. The OCT fiber catheter was firmly clamped to the micro drive equipped stereotaxic frame and positioned on top of the burr hole. The microelectrode shank was placed between the exposed skull and the OCT fiber precisely aligned above the hole (Figure 3A). Slowly driving the stereotactic z arm to insert the fiber catheter, the flexible microelectrode was pushed into the brain by the fiber catheter (Figures 3B,C). We stopped the insertion when the fiber catheter was at 6 mm under the brain surface (thalamic nucleus area) (Figure 3D). In order to avoid electrode displacement while applying dental cement, a small drop of Super Glue (Loctite 4061, Henkel Loctite GmbH) was applied to adhere the rest of the electrode shank to the skull. A small amount of bone wax was plastered to close the skull burr hole. We sealed and tethered the connector part of the fiber catheter and the microelectrode with adequate dental cement (Figure 2A). The skin incision was sutured to close and wiped with betaisodona (7.5%, B. Braun Melsungen) to prevent skin infection. Carprofen (4 mg/kg) was then administered subcutaneously for pain management each day for 5 days after surgery. The animals were housed with daily inspection.

### 2.5. Post surgery measurement and OCT signal analysis

The OCT signal was collected at week 1, 2, 3, 6, and 12 after the implantation surgery in freely moving rats. During each measurement, the implanted OCT fiber catheter was connected to the OCT sampling cable through FC/FC mating sleeve. Since the FC ferrule features a flat endface, the presence of air at the junction point induces significant signal loss due to the refractive index mismatching between air and fiber material. To achieve the maximum light transmission between the fiber catheter and the sampling cable, we applied a thin layer (~100 μm thick) of index matching gel (G608N3, Thorlabs) at the junction part. Signal acquisition was performed for about 5 min in each measurement section for analysis.

**Figure 4:**
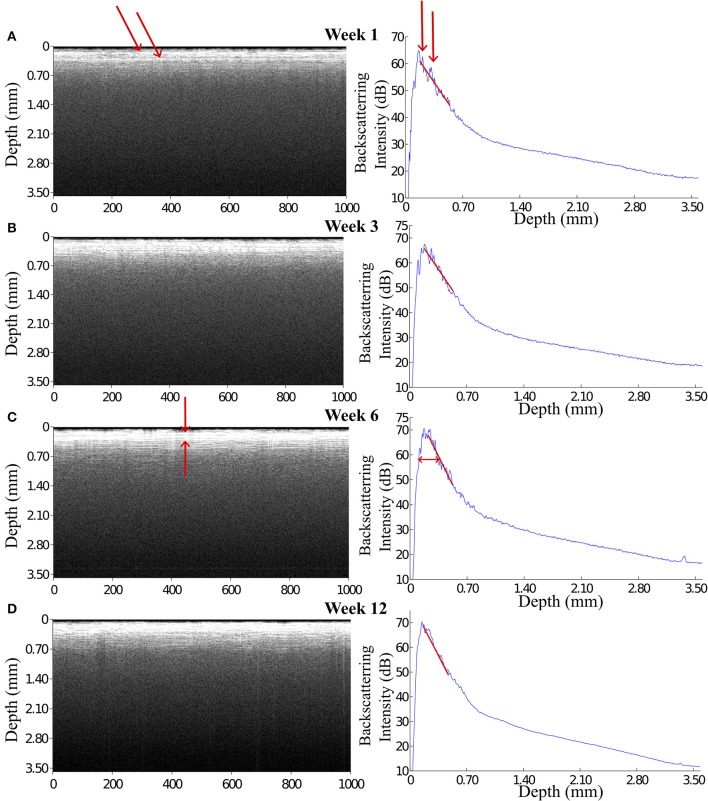
**OCT images and the corresponding A-scan profiles acquired at week 1, 3, 6, and 12 after implantation**. The intensity of the backscattered light increased during the first 3 weeks post implantation **(A,B)**, while at week 6 and 12 the OCT signal attenuated faster featuring a steep declining slope in the A-scan plot **(C,D)**. Arrows in panel **(A)** indicate the anatomic micro structures viewed by the OCT. Arrows in panel **(C)** indicate a 200 μm thick layer is expressed in the OCT signal.

The OCT signal is presented in gray scale images each of which consists of 1000 A-scan signals aligned in x-axis (see Figure 4 left panels). Depth-scan profile is plotted by taking an average of 1000 A-scans (see Figure 4 right panels). For quantification of OCT signals, we calculated the light attenuation factor of the sampled tissue by analysing A-scan signals. The light attenuation factor was defined as the ratio of detected light intensity decline in depth (mm). It is proportional but not equal to the tissue attenuation coefficient which is one of the essential tissue optical properties reflecting the tissue composition and density. A change in attenuation factor implies alterations in tissue properties, such as cell density, cell orientation, predominate cell type and hence backscatter ability. Attenuation factor is calculated as the slope value of the linear fit for the log intensity of the linear portion depth in an A-scan plot (see the red lines on plots in Figure 4).

### 2.6. Histology and immunostaining

Rats were terminated by administering Ketamine and Xylazine mixture intraperitonealy. The brains were removed immediately after decapitation, fixed in 4% paraformaldehyde (PFA) in PBS for 4 days, and then incubated in 30% sucrose in PBS for at least 2 days. During the brain removal procedure, the flexible microelectrode and the OCT fiber probe were carefully cut from their skull-tethered part and were PFA fixed within the brain. Prior to sectioning, the OCT fiber probe was explanted from the brain while the flexible microelectrode was kept *in situ* in the brain. Brains were then embedded in Tissue-Tek O.C.T. (Sakura Finetek, Germany) at −20°C. 20 μm thick transversal or coronal slices were obtained with a cryostat-microtome at −20°C.

Brain slices were double-immunostained for GFAP (glial fibrillary acidic protein) to detect astrocytes and Iba1 (ionized calcium-binding adapter molecule (1) to identify microglia/macrophages. Brain slices were incubated in 10% goat serum to block non-specific binding, then incubated in GFAP antibody (1:1000 dilution, rabbit IgG, Millipore) and Iba1 antibody (1:500 dilution, Goat IgG, Abcam) for 3 h. After 3 rinses in PBS (5 min each), fluorescence-labeled secondary antibodies AlexaFluro 488 anti-rabbit and AlexaFluro 647 anti-goat were applied for 1 h. Both secondary antibodies (Molecular Probes) were used in 1: 1000 dilution. After 3 rinses in PBS (5 min each), brain slices were mounted onto the microscopic slides with Dapi-Fluoromount-G (Southern Biotech) and then covered with a coverslip. Slice images were collected with Jenoptik camera mounted on a Zeiss microscope (Zeiss Imager a1), using the identical exposure time for each immunolabel at each magnification.

## 3. Results

### 3.1. OCT signals from the tissue around the indwelling fiber catheter

**Figure 5:**
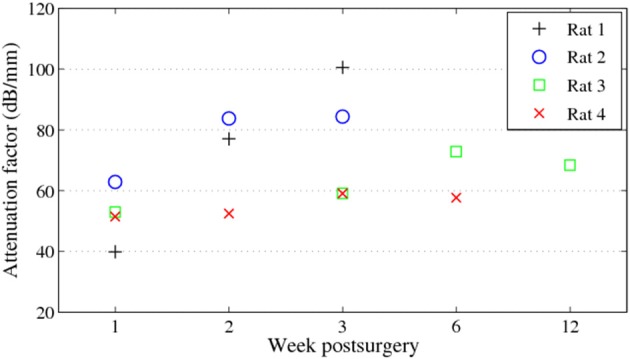
**Attenuation factors of the brain tissue around the fiber catheter at week 1, 2, 3, 6, and 12**.

During 6 weeks after the implantation, the intensity of the OCT signal increased from 60 to 70 dB (Figures 4A–C). From week 6 to week 12, the OCT signal intensity didn't demonstrate noticeable change (Figures 4C,D). Some sparsely distributed microscopic structures could be identified close to the fiber catheter 1 week post operation, having the shape of as several horizontal stripe patterns and appear in the A-scan signal as multiple peaks (Figure 4A). At week 6, a 200 μm thick layer of tissue with high backscattering signal had developed around the fiber catheter (Figure 4C). This high reflecting tissue layer developed into a prominent feature in the OCT image at week 12, with a smooth A-scan plot profile (Figure 4D). At week 6 and 12, the OCT signal attenuated faster featuring a steep declining slope in the A-scan plot while the backscattering intensity from the tissue surrounding the fiber probe stayed steady at 70 dB (see Figures 4C,D). To further characterize the OCT signal, the attenuation factor of 4 animals on each measurement were calculated and presented in Figure 5. In general, the attenuation factor evidently raised during the first 3 weeks after the implantation, implying that a compact dense tissue layer was developing during this period. Then the attenuation factor stabilized at 6 weeks and 12 weeks post implantation.

### 3.2. OCT signals of the indwelling flexible microelectrode

**Figure 6:**
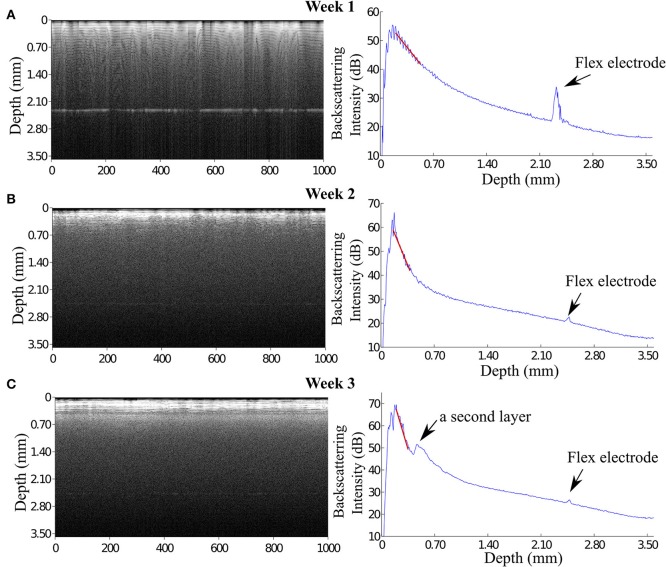
**OCT images reveal that the signal of flexible microelectrode underneath the fiber catheter declined**. **(A)** At 1 week, the flexible electrode can be seen distinctly in the OCT image with a approximately 12 dB peak height in the A-scan plot. **(B)** In contrast to the enhanced OCT signal of the brain tissue, the signal of the flexible electrode severely declined. **(C)** Interestingly, at 3 weeks p.i. the OCT image illustrates 2 distinguishable tissue layers. The backscattering intensity of the flexible electrode remains noticeable.

At week 1 post implantation, the signal from the flexible probe was evidently visible in the A-scan signal located at 2.3 mm underneath the OCT fiber catheter tip. The maximum backscattering intensity of the OCT signal at week 1 post implantation (p.i.) is about 59 dB, while the backscattering intensity from the flexible electrode is approximately 35 dB presenting a a clearly visible peak (Figure 6A). Since before it reached the flexible probe the incident light was presumably scattered and attenuated by the ongoing scar formation around the glass fiber catheter, the backscattered signal from the flexible electrode dwindled in the following weeks (Figures 6B,C). Although the incident light attenuated predominantly in the tissue around the fiber presumably the scar sheath, the flexible electrode was still visible in the OCT scan demonstrating as a noticeable peak in the A-scan plot.

**Figure 7:**
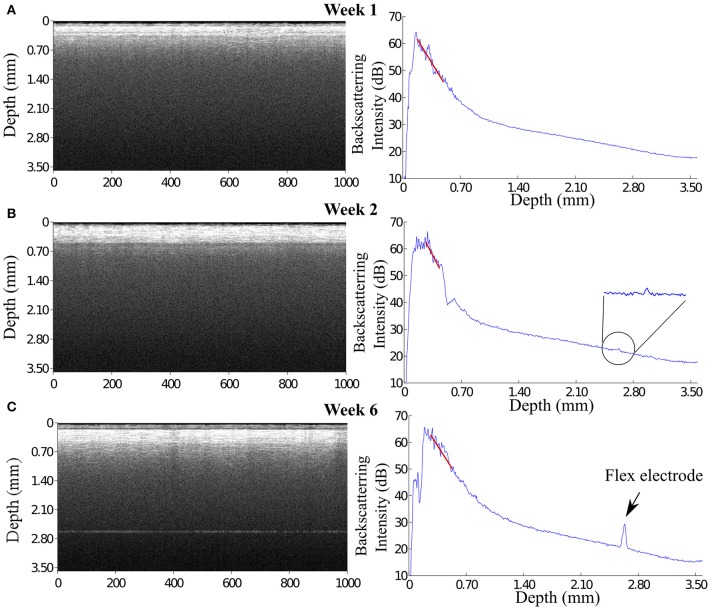
**(A)** At 1 week, the flexible electrode can not be seen in the OCT image probably because of a misalignment between the flexible electrode and the fiber catheter. **(B)** At 2 weeks, a small but noticeable peak appears in the A-scan plot at the depth position of 2.70 mm. **(C)** At 6 weeks, a pronounced signal is revealed both in the OCT image and in the A-scan plot, presumably, from the flexible electrode.

Although the signal of the flexible electrode was expected to diminish due to the strong light scattering in the compact glial sheath around the rigid fiber catheter, we observed in one case that the signal of the flexible electrode reappeared at 6 weeks p.i. (Figure 7). This very flexible probe was not visible after the first week, presumably as a result of the incorrect alignment of the flexible electrode and the fiber catheter upon implantation (Figure 7A). At week 2 p.i. in addition to the two-layer structure formation at the depth position of around 0.60 mm in the OCT image, a small peak was observed in the A-scan plot at the depth position of 2.70 mm (Figure 7B). At week 6 p.i. a pronounced signal at the depth position of 2.70 mm was detected and visible both in the OCT image and the A-scan plot (Figure 7C). We assume, that the high intensity signal that reappeared at the image depth of 2.70 mm at 6 weeks p.i. is originating from the flexible electrode. Since this polyimide-based electrode features much lower elastic modulus compared to the conventional metal microelectrode with similar dimensions, the flexible electrode is supposedly able to float with the brain tissue (Jensen et al., 2007). Therefore, during the 6 weeks the flexible electrode's position in the brain might alter and move back to the detection area of the OCT fiber catheter eliciting the high intensity peak in OCT signal.

### 3.3. Immunoreactivity of GFAP positive cells and Iba-1 positive cells around the insertion trajectory

**Figure 8:**
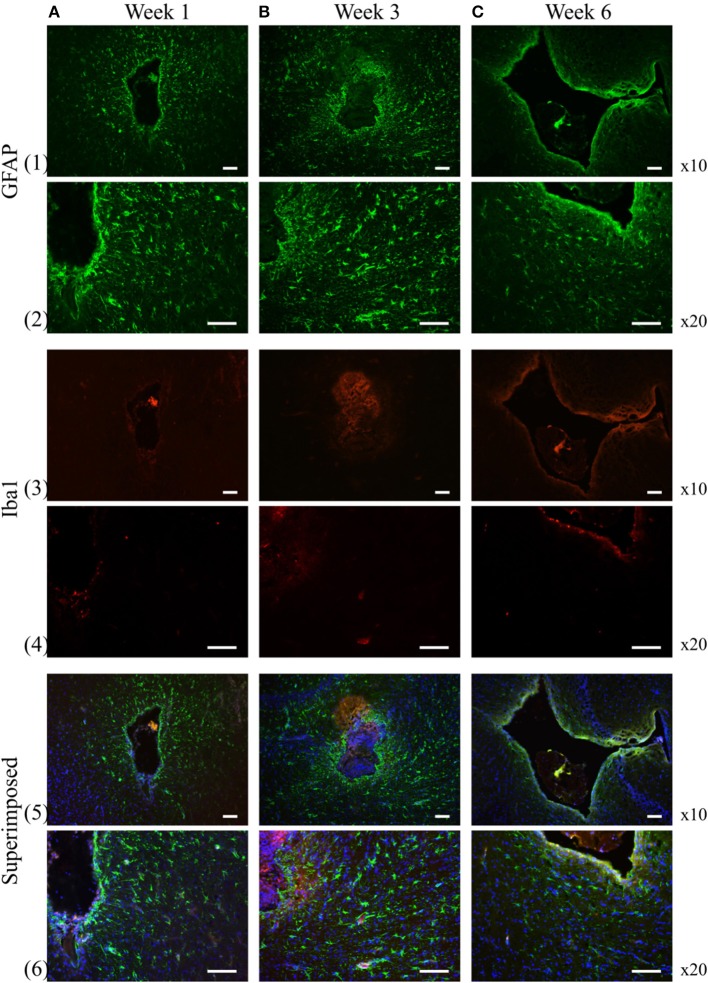
**GFAP and Iba-1 reactivity to the chronically implanted fiber catheter and flexible electrode assembly at week 1, 3, and 6, respectively**. At week 1 and 3 the GFAP positive cells spread to larger area around the insertion trajectory **(1A,B, 2A,B)**, while at week 6 the GFAP positive cells become less pronounced in the extended surrounding area, instead they construct a compact layer next to the electrode and fiber **(1C, 2C)**. Iba-1 positive cells are much smaller numbers than the GFAP positive cells at all time points **(3A–C, 4A–C)**. No obvious proliferation of the Iba-1 positive cells are observed over time. Row 5 and 6 are the superimposed pictures of GFAP (green), Iba-1 (red) and DAPI (blue) staining. Scale bar = 100 μm.

The reactivity of GFAP-positive cells are widely used to characterize astrocytes star-shaped cell body (Schiffer et al., 1993; Faulkner et al., 2004; Sofroniew, 2005). At week 1, GFAP-positive cells are loosely distributed in an area up to 500 μm from the insertion cavity. A thin layer of the aligned GFAP-positive cells was observed surrounding the insertion channel. At week 3, GFAP-positive cells proliferated and spread to further areas up to 700 μm from the insertion edge with enhanced fluorescence intensity. At week 6, the fluorescence of GFAP-positive cells in the far area appeared reduced. Instead, a layer GFAP-positive cells with high fluorescence intensity encapsulated the insertion site (Figure 8).

We assessed the inflammatory process during the experiment by analyzing the expression of the ionized calcium binding adaptor molecule (Iba-1), a marker for both resting and reactive microglia and macrophages (Ito et al., 1998). At 1, 3, 6 weeks, the Iba-1 expression was only found in the close area of the insertion site not in the extended surrounding area (Figure 8). The apparent distribution of fluorescence intensity and the number of the Iba-1 positive cells didn't demonstrate any significant changes over time.

## 4. Discussion

**Figure 9:**
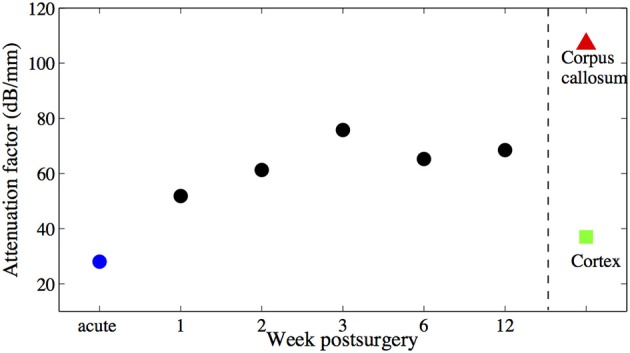
**Averaged attenuation factors of the brain tissue around the fiber catheter at week 1, 2, 3, 6, and 12 are marked with black •, attenuation factor obtained from acute experiment is marked with blue •**. Attenuation factors of other brain tissue types obtained also in acute experiments (Xie et al., 2013) are marked with ∆ and □ respectively. No error bars are shown, due to the littel number of animals used.

The goal of this study is to introduce a new method to monitor and assess the scarring process around a chronically implanted glass fiber catheter and a synchronously implanted flexible polyimide-based microelectrode by using an OCT imaging system (catheter type, ø125 μm). OCT signals were acquired weekly for 12 weeks after implantation even though no technical obstacles prohibit continuous monitoring. The results corroborate that SDOCT with an *in situ* implanted fiber catheter is capable of visualizing the modification in optical properties of the surrounding tissue over time. During the first 3 weeks after implantation, the intensity of the backscattered light, hence the OCT signal of the surrounding tissue increases monotonically. The intensity levels off at 6 weeks after implantation. The increased backscattering intensity from the surrounding tissue is most likely caused by the increased accumulation of astrocytes around the glass fiber itself, which is referred in literature as glial encapsulation (Leach et al., 2010). The analyzed tissue optical property “attenuation factor” demonstrates that a compact dense tissue sheath has been developing during the 6 weeks after implantation. Since we've obtained the attenuation factor of control brain tissue from a previous study, we are able to compare the current result to naive control tissue (Figure 9). Besides, it is promising that within the proposed novel monitoring modality (1) the 10 μm thick flexible probe is still visible up to 2.7 mm away from the fiber tip, (2) alteration in tissue anatomical structure is noticeable in OCT A-scans, such as the two-layered structure that developed from week 3 on in (Figure 6).

Since the OCT fiber cannula itself is recognized as a foreign body by the brain tissue, the incident light is scattered and attenuated already at the compact astrocyte sheath around the fiber tip, resulting in a deteriorated detection of the flexible probe, but at the same time providing positive control of immune response. This shortcoming could be overcome by using a probe assembly which consists of a waveguide (for OCT acquisition) that is integrated to a flexible polyimide probe (for electrophysiology acquisition) to gain “inside-out” views of the developing gliosis while maintaining the probe's flexibility.

Still, it must be noted, that the flexible probe remained visible even at distances greater than 1 mm without revealing a change in tissue optical properties. It is a matter for ongoing investigations, whether the flexible probe was indeed not covered by a detectable glial sheath as independent immunocytochemistry results suggest (unpublished data) or whether an existing sheath did not sufficiently backscatter for detection. In any ways, to the best of our knowledge, the presented method appears to be the first optical attempt to monitor brain's immune response on deep implants without prior markers *in vivo*.

### Conflict of interest statement

The authors declare that the research was conducted in the absence of any commercial or financial relationships that could be construed as a potential conflict of interest.
